# What do we know about the impact of sexual violence on health and health behaviour of women in Estonia?

**DOI:** 10.1186/s12889-020-09953-2

**Published:** 2020-12-10

**Authors:** Hedda Lippus, Made Laanpere, Kai Part, Inge Ringmets, Helle Karro

**Affiliations:** 1 Department of Obstetrics and Gynaecology, Institute of Clinical Medicine, University of Tartu, 8 Puusepa, 51014 Tartu, Estonia; 2 Hubert Department of Global Health, Rollins School of Public Health, Emory University, Atlanta, GA USA; 3 Sexual Health Clinic of Tartu, Tartu, Estonia; 4 Tartu University Hospital Women’s Clinic, Tartu, Estonia; 5 Institute of Family Medicine and Public Health, University of Tartu, Tartu, Estonia

**Keywords:** Sexual violence, Women’s health, Risky health behaviours, Risky sexual behaviours

## Abstract

**Background:**

Sexual violence against women is a major public health issue and a breach of human rights. Although various consequences of sexual violence on health have been described in a large number of scientific publications, very little is known about this topic in Estonia. The aim of this study was to examine the prevalence of sexual violence and associations between exposure to sexual violence and risky health and sexual behaviours among women in Estonia.

**Methods:**

A population-based cross-sectional study was carried out in Estonia in 2014. Self-reported data regarding selected indicators of risky health and sexual behaviours were collected from 1670 women, aged 18–44 years, via a self-administered questionnaire. To measure the prevalence of sexual violence, questions from the NorVold Abuse Questionnaire were included. Chi-square and multivariate logistic regression were used to analyse the data.

**Results:**

Of the respondents, 22.7% (*n* = 379) reported being exposed to sexual violence during their lifetime, and over half of these women had had these experiences before the age of 18. Statistically significant associations were found between sexual violence and smoking (adjusted odds ratio (AOR) 1.32, 95% CI 1.03–1.70), alcohol consumption (AOR 1.52, 95% CI 1.18–1.95), illicit drug use (AOR 2.21, 95% CI 1.70–2.89), sexual intercourse for money or other material reward (AOR 3.51, 95% CI 1.62–7.61), concurrent sexual relationships (AOR 2.64; 95% CI 1.80–3.86), and being diagnosed with sexually transmitted infections (AOR 1.48, 95% CI 1.09–2.01).

**Conclusions:**

In Estonia, sexual violence against women is widespread and is associated with several risky health and sexual behaviours. Efforts should be made, both among the general public and professionals, to raise awareness regarding the prevalence and negative impact of sexual violence. Women who have been exposed to sexual violence are in need of professional medical, legal and psychological help free from prejudice to help them recover from such traumatic events.

## Background

The World Health Organisation defines sexual violence (SV) as follows: “Any sexual act, attempt to obtain a sexual act, unwanted sexual comments or advances, or acts to traffic or otherwise directed against a person’s sexuality using coercion, by any person regardless of their relationship to the victim, in any setting, including but not limited to home and work” [1]. By definition, SV involves a party who does not give or is unable to give consent.

There is a significant amount of evidence, demonstrating that SV can lead to a variety of negative immediate and long-term health consequences and it is known to be associated with risky health and sexual behaviours [1–9]. Women exposed to SV use illicit drugs, smoke and binge drink more often, possibly to reduce the negative feelings associated with SV [7, 10, 11].

SV is also associated with a higher prevalence of sexually transmitted infections (STIs), including HIV, and engaging in sex without using contraception [7, 12, 13]. SV can increase the risk of contracting a STI directly (coerced unprotected sex with an infected person) or indirectly through an increase in risky sexual behaviour (e.g., condom non-use, having concurrent sexual relationships) [3, 9, 14].

For many reasons, SV has been underreported and not widely recognized in Estonia. Violence against women is rooted in gender inequality, which remains a problem in Estonia [15, 16]. For example, gender pay gap in Estonia is over 22% – the highest in the European Union (EU) [17]. In addition to that, victim-blaming attitudes, which have been shown to play a paramount role in higher prevalence of violence against women, are common in Estonia [18]. A study carried out in 2010 among 27 member states of the EU showed that 84% of the respondents in Estonia considered the behaviour of women as a predisposing factor to violence against them, which was the second highest result [19]. Although a survey conducted by the European Agency for Fundamental Rights in 2014 revealed that 13% of women in Estonia have been exposed to SV by a partner or non-partner starting from the age of 15, only 11% of women in Estonia thought that violence against women in their country was very common, compared to the EU average of 27%. As these studies suggest, gender inequality and low awareness about violence against women remain serious but underexplored issues in Estonia.

While in western countries the relationship between gender inequality and violence against women has been a topic of academic research for decades, it has been neglected in Estonia. Estonia was occupied by the Soviet Union for half of a century and regained its independence in 1991. Although the Soviet Union had its policies that seemingly promoted gender equality, violence against women was often ignored and remained undocumented [20]. More recently, however, there has been a steady increase in research looking at SV and other forms of violence against women [21, 22]. Nevertheless, the impact of SV on Estonian women’s health and sexual behaviours has not been studied, and the current study seeks to contribute to closing this gap. The aim of this study was to examine the prevalence of SV in Estonia and its associations with risky health behaviours (smoking, excessive alcohol consumption and illicit drug use) as well as risky sexual behaviours (contraception non-use during the most recent sexual intercourse, having sexual intercourse for money or other material reward, concurrent sexual relationships, and contracting STIs).

## Methods

### Sample and sampling

The study was based on a Women’s Health Survey conducted in Estonia between March and August 2014. The aim of the survey was to collect information about various sexual and reproductive health indicators of women aged 16–44 which are not routinely collected (e.g. the use of contraceptive methods, reproductive plans, the prevalence of infertility and violence, sexual health care services etc.). A random sample of the female population, stratified by age groups (16–17, 18–24, 25–34, and 35–44), was taken from the Population Registry. For sample size calculations, data from previous studies [23, 24] regarding the percentage of sexually active respondents in each age group and total response rates were taken into account in order to get a sufficiently large sample. The initial sample size was 5233 women, and by age group the numbers were 2112 (16–17), 1144 (18–24), 993 (25–34), and 984 (35–44). For this calculation, the OpenEpi software package [25] was used.

Of the initial sample, 40 women were not able to reply. Out of 5193 eligible respondents, 2708 did not return the questionnaire, and 12 refused to answer. In addition, 24 questionnaires were returned with very few answered questions, six had conflicting answers and three respondents were over 45 years old. The final response rate was 47.0%. Of the respondents, 16.6% answered electronically and 83.4% on paper. The survey methods are described in more detail in the study report, where the full questionnaire in English is also included [26].

For this paper, women aged 16–17 years and women who had not answered to the question about age were left out of the analysis (*n* = 727). Respondents aged 16–17 years were not included due to the nature of the questions used to determine risky sexual behaviour, as only 50.6% in that age group had experienced sexual intercourse. Of the respondents, 37 women did not answer the questions about SV and six were left out of the analysis because their native language was neither Estonian nor Russian. The final sample size was 1670.

### Survey

The questionnaire contained questions about socio-demographic background, intimate relationships and sexuality, reproductive history, satisfaction with health care services, contraception, any future plans of having children, overall health, and exposure to violence.

### Sexual violence assessment instrument

To assess exposure to SV we used NorVold Abuse Questionnaire (NorAQ), which has good reliability and validity [27]. In the analysis for this paper we used four questions from the NorAQ:

1. “SV with no genital contact”: Has anybody against your will touched parts of your body other than the genitals in a ‘sexual way’ or forced you to touch other parts of his or her body in a ‘sexual way’?
2. “Sexual humiliation”: Have you in any way been sexually humiliated; e.g., by being forced to watch a porno movie or similar against your will, forced to participate in a porno movie or similar, forced to show your body naked or forced to watch when somebody else showed his/her body naked?
3. “SV with genital contact”: Has anybody against your will touched your genitals, used your body to satisfy him/herself sexually or forced you to touch anybody else’s genitals?
4. “Rape/attempted rape”: Has anybody against your will put his penis into your vagina, mouth or rectum or tried to put an object or other part of the body into your vagina, mouth or rectum?

For each question the respondent could choose between the answers: no; yes, as a child (less than 18 years old); yes, as an adult (18 years old or older); yes, as both a child and an adult. We created an aggregated measure where all women who gave an affirmative answer to at least one of the questions about SV were considered to have been exposed to SV.

### Socio-demographic characteristics

Self-reported socio-demographic factors were used as categorical variables with following categories: three groups based on age (18–24; 25–34; 35–44 years); two groups based on the language spoken (Estonian; Russian); three groups based on the level of education (basic or less; secondary/vocational secondary; vocational higher/bachelor’s degree/master’s or doctoral degree); three groups based on marital status (married/cohabiting; separated/divorced/widowed; single); four groups based on employment status (employed; pupil/student/postgraduate student; on pregnancy or parental leave; retired/disability pension); and three groups based on the reported level of difficulties in paying bills (always/often; sometimes; rarely/never).

### Risky health and sexual behaviour variables

Due to the nature of binary multiple logistic regression analysis all outcome variables were dichotomised.

To determine risky health behaviour, smoking (never a smoker; past/current smoker), drinking enough alcohol to lose control of oneself (daily/weekly/monthly/less often than monthly; never) during the last 12 months, and ever using illicit drugs (no; yes) were considered in analysis.

We used four questions to evaluate risky sexual behaviour. To determine contraception use during the most recent intercourse, the respondents could choose a contraceptive method from a list or they could answer that they did not use any method. When analysing this question, we left out women who did not need contraception (were pregnant, wanted to get pregnant, or were breastfeeding a less than 6-month-old baby; *n* = 169). Participants were asked “Have you been asked to have sex in exchange for money or other material reward?”, and respondents were assigned into two groups: the first group consisted of women who had not been asked to do so and also of those who had been asked, but refused and to the second group women who had accepted the offer. To assess whether the respondent had concurrent sexual relationships, the following question was asked “Have you had concurrent sexual relationships during your present marriage/cohabitation”, and responses were categorized into two groups (yes; no). Women who were not currently in a relationship were left out of the analysis (*n* = 191). We asked if the respondent had ever been diagnosed with at least one of the following STIs: chlamydia; gonorrhoea; trichomoniasis; HIV/AIDS; syphilis. The respondents were categorized into two groups (yes; no). Respondents who did not know whether they had ever been diagnosed with an STI or had not been tested, were not included into analyses that involved this variable (*n* = 116).

### Analysis

Differences between women exposed to SV and not exposed to SV were analysed using a chi-square test with a significance level of *p* < 0.05. Selected socio-demographic potential confounders (age, education, marital status, occupation, difficulties with paying bills) were entered into multivariable binary logistic regression analysis models to explore associations between SV exposure and risky health and sexual behaviours. Associations are presented as odds ratios (OR) and adjusted odds ratios (AOR) with 95% confidence intervals (CI 95%).

## Results

In our study, 22.7% (*n* = 379) of respondents reported having been exposed to SV during their lifetime: 18.1% to SV with no genital contact, 13.4% to SV with genital contact, 9.2% to rape/attempted rape and 4.0% to sexual humiliation. Table 1 shows the distribution of women who had been exposed to SV by age during the exposure (before the age of 18, being 18 years old or older, and both) for four types of SV. Most of the women who had been exposed to SV, with exception of rape/attempted rape, were exposed before the age of 18. Among women who had been exposed to SV, approximately 10% had been exposed to it before and after the age of 18 across all four types of SV.

**Table 1:** The distribution of age during the exposure by the type of sexual violence (SV) among women aged 18–44 years in Estonia who have been exposed to SV, *n* (%)

Type of SV	Before the age of 18 years	Being 18 years old or older	Before and after the age of 18	Total
	(*n* = 206)	(*n* = 136)	(*n* = 37)	(*n* = 379)
SV with no genital contact	180 (59.6)	97 (32.1)	25 (8.3)	302 (100)
Sexual humiliation	45 (68.2)	14 (21.2)	7 (10.6)	66 (100)
SV with genital contact	127 (57.0)	73 (32.7)	23 (10.3)	223 (100)
Rape/attempted rape	66 (43.1)	76 (49.7)	11 (7.2)	153 (100)

Selected self-reported socio-demographic characteristics according to exposure to SV are shown in Table 2. As expected, among women in the oldest age group, the proportion of those who had been exposed to SV was the highest. SV was also more frequent among women who were separated/divorced/widowed, did not currently work (housewives, unemployed, retired or on a disability pension) and/or had always or often difficulties with paying bills. There were no significant differences between Estonian- and Russian-speaking respondents, nor between women of different levels of education.

**Table 2:** Selected socio-demographic characteristics by sexual violence (SV) exposure, for women aged 18–44 years in Estonia, *n* (%)

Socio-demographic characteristics	Total	SV exposure during lifetime		p value^a^	Effect size^a^
		No	Yes		
	*n* = 1670	*n* = 1291	*n* = 379		
**Age in years**				< 0.001	0.14
18–24	724	604 (84.4)	120 (16.6)		
25–34	473	361 (76.3)	112 (23.7)		
35–44	473	326 (68.9)	147 (31.1)		
**Native language**				0.981	
Estonian	1315	1018 (77.4)	297 (22.6)		
Russian	340	263 (77.4)	77 (22.6)		
Missing	15	10 (66.7)	5 (33.3)		
**Education**				0.583	
Basic or less	383	293 (76.5)	90 (23.5)		
Secondary/vocational secondary	695	532 (76.6)	163 (23.5)		
Vocational higher/bachelor’s/master’s /doctoral degree	584	460 (78.8)	124 (21.2)		
Missing	8	6 (75.0)	2 (25.0)		
**Marital status**				0.002	0.09
Married/cohabiting	1078	816 (75.7)	262 (24.3)		
Separated/divorced/widowed	71	47 (66.2)	24 (33.8)		
Single	510	418 (82.0)	92 (18.0)		
Missing	11	10 (90.9)	1 (9.1)		
**Occupation**				< 0.001	0.11
Employed	852	636 (74.7)	216 (25.4)		
Pupil/student/postgraduate student	496	417 (84.1)	79 (15.9)		
On pregnancy/parental leave	172	130 (75.6)	42 (24.4)		
Housewife/unemployed/retired/disability pension	115	79 (68.7)	36 (31.3)		
Missing	35	29 (82.9)	6 (17.1)		
**Difficulties paying bills**				0.001	0.09
Always/often	166	112 (67.5)	54 (32.5)		
Sometimes	341	252 (73.9)	89 (26.1)		
Rarely/never	1143	910 (79.6)	233 (20.4)		
Missing	20	17 (85.0)	3 (15.0)		

^a^Chi-Square and Cramér’s V test were applied only for non-missing values

Table 3 presents the odds ratios and adjusted odds ratios of selected risky health and sexual behaviour outcomes for the women who had been exposed to SV and for those who had not been exposed. Statistically significant associations between SV and smoking, alcohol consumption, illicit drug use, sexual intercourse for money or other material reward, having concurrent sexual relationships, and being diagnosed with STIs were found. Contraception non-use during their most recent intercourse was significantly associated with being exposed to SV only before the adjustment.

**Table 3:** The prevalence, odds ratios (OR) and adjusted odds ratios (AOR) of selected indicators of risky health and sexual behaviour associated with exposure to sexual violence (SV), for 18–44 years old women in Estonia

Risky health and sexual behaviour	Total (*n*)	No exposure to SV *n* (%^a^)	Exposure to SV *n* (%^b^)	OR (95% CI)	AOR^c^ (95% CI)
Smoking	1596	482 (39.1)	170 (47.0)	1.38 (1.09–1.75)	1.32 (1.03–1.70)
Alcohol consumption	1599	588 (47.6)	191 (52.5)	1.22 (0.96–1.54)	1.52 (1.18–1.95)
Illicit drug use	1596	303 (24.6)	137 (37.5)	1.85 (1.44–2.37)	2.21 (1.70–2.89)
Contraception non-use	1439	104 (9.5)	47 (13.9)	1.55 (1.07–2.24)	1.26 (0.86–1.85)
Sexual intercourse for money/ material reward	1601	12 (1.0)	18 (4.9)	5.27 (2.51–11.05)	3.51 (1.62–7.61)
Concurrent sexual relationships	1165	80 (9.0)	58 (21.1)	2.71 (1.87–3.92)	2.64 (1.80–3.86)
STIs	1485	190 (16.5)	94 (28.0)	1.96 (1.48–2.61)	1.48 (1.09–2.01)

^a^Percentage of risky health/sexual behaviour among all women with no exposure to SV

^b^Percentage of all risky health/sexual behaviour among women with exposure to SV

^c^Adjusted for age, marital status, education, occupation, and difficulties with paying bills

## Discussion

Regarding the prevalence of SV, our findings are consistent with previous studies carried out in Estonia [28, 29]. In a survey among pregnant women in Estonia, where similarly to this study the NorAQ questionnaire in Estonian and Russian was used, 18% of the respondents reported exposure to SV during their lifetime [29]. To the best of our knowledge, this is the first population-based study in Estonia to demonstrate a higher prevalence of risky health and sexual behaviour among women exposed to SV. We found an increased risk for smoking, alcohol consumption, using illicit drugs, agreeing to have sexual intercourse for money or other material reward, having concurrent sexual relationships during their marriage or cohabitation and being diagnosed with STIs. These results provide further evidence that SV against women in Estonia is a significant problem.

Our study shows that most of the women who had been exposed to SV, were exposed to it before the age of 18. Concerning ethnic background, no differences between the Estonian- and Russian-speaking women in terms of SV exposure during lifetime were found in this study. In Estonia, there is a large ethnic minority of Russians, who make up 25% of the population [30]. Previous studies in Estonia have found that attitudes and knowledge concerning SV among the Estonian- and Russian-speaking population are different: among Russian-speaking respondents victim-blaming is more common and fewer violent acts are recognized as violence (e.g., considering forced sexual intercourse in a relationship to be a form of SV) [13, 18, 21, 31]. One possible explanation for the results in the current study is that Russian-speaking women underreported exposure to SV.

In this study no significant differences were found in the proportion of women exposed to SV among groups with different educational levels, which is in accordance with the EU wide survey from 2014 [28]. This finding adds further evidence that women at all levels of educational attainment suffer from SV.

Our findings demonstrate the broad scope of risky health and sexual risk behaviour associated with SV. Alcohol and illicit drug use have been associated with an increased risk of becoming exposed to SV, as intoxication makes it is more difficult for women to protect themselves [4, 32]. However, increased alcohol and illicit drug use after exposure to SV has also been demonstrated [10, 33]. Excessive alcohol consumption, illicit drug use and smoking have been associated with the wish to reduce the symptoms of depression and post-traumatic stress disorder linked to SV [11, 33]. In this study we saw statistically significant differences in smoking, illicit drug use and alcohol consumption between women who had been exposed to SV and those who had not.

Our results provide further evidence that women who have been exposed to SV are more likely to engage in risky sexual behaviour [34, 35]. They have more concurrent sexual relationships, engage in sexual relationships for money or other material reward more often, and are less likely to use contraception. This in turn increases the risk of unwanted pregnancy and STIs, including HIV. There are probably multiple different pathways through which SV leads to risky sexual behaviour. Possible explanations could be that risky sexual behaviour is mediated through illicit drug and alcohol abuse, which causes impaired judgment and risk negotiation abilities [11, 35]. Among some women the need to find ways to support the drug habit, which may have appeared or become more severe after exposure to SV, can lead to having transactional sex [36]. Another explanation could be that SV has severe psychological effects that can lead to an overall neglect of one’s health and avoidance of thinking about the potential negative consequences of risky behaviour [33–35]. Interestingly there are data suggesting that some women engage in transactional sex after exposure to SV as a way of regaining control over their bodies [36].

Our study had some limitations that should be pointed out. Due to the cross-sectional study design, it was not possible to determine whether risky health and sexual behaviour preceded or followed exposure to SV. Based on previous evidence we presume that risky behaviour is a consequence of SV rather than a factor that is conducive to exposure to SV [3]. Longitudinal studies are needed to examine the chronological relationship between SV and risky health and sexual behaviour. Also, there may be a recall bias, as violent events may be suppressed and thus not reported. In addition to that, the recall-rates may not have been equal across the four SV categories included in our study. We can only hypothesize, that the recall-rate might have been higher for the most serious events e.g. rape. However, it should be pointed out that the response rate was in line with our calculations regarding sample size and study power, and very few respondents did not answer the questions concerning SV, depending on the question it stayed between 1.4 and 2%. Lastly, the subgroup of women who had agreed to have sex for money or other material reward was relatively small, which leads to the less precise estimates. Although transactional sex is legal in Estonia, it is still highly stigmatized, which could have caused underreporting [37]. However, it should be noted, that findings from previous studies support the association between exposure to SV and having sex for money or other reward demonstrated in this study [36, 38].

## Conclusions

The high prevalence of SV, including the high rate before the age of 18, should be acknowledged as a major public health problem. Changes in public beliefs and practices are a prerequisite for preventing SV. All healthcare professionals should be educated about the possible associations between certain health problems and exposure to SV, so that they would recognize it and know how to react once violence has been detected. In order for the healthcare professionals to be able to provide the best possible care and necessary services, they have to be supported by the healthcare system by for example creating effective violence screening systems, allowing time to do the sensitive work and having efficient referral systems [39]. Survivors of SV should be encouraged to seek professional help, which includes assuring that provided help is free from prejudice to prevent revictimization. The goal when SV has occurred should be early intervention to prevent risky health and sexual behaviour.

Our results help to draw attention to the current situation concerning SV and its impact on health and health behaviour in Estonia. Since year 2016 government- funded sexual assault centres have been founded in Estonia, being the first ones in the former Soviet countries and Eastern-Europe. Findings from this study can be used for the further development of the services offered in these centres. This information is essential for understanding and noticing the needs of SV survivors to prevent negative health sequel. In addition to Estonia, this data can be used in other countries, especially in those with similar background and current socio-economic situation.

## Data Availability

The dataset analysed during the current study available from the corresponding author on reasonable request.

## Abbreviations

SV: Sexual violence
STI: Sexually transmitted infection
NorAQ: NorVold Abuse Questionnaire
EU: European Union
